# Electrical Properties of Thin-Film Capacitors Fabricated Using High Temperature Sputtered Modified Barium Titanate

**DOI:** 10.3390/ma5040644

**Published:** 2012-04-13

**Authors:** Glyn J. Reynolds, Martin Kratzer, Martin Dubs, Heinz Felzer, Robert Mamazza

**Affiliations:** 1 Oerlikon USA, Inc., Business Unit Systems, 970 Lake Carillon Dr, Suite 300, St. Petersburg, FL 33716, USA; 2 OC Oerlikon Balzers AG, Business Unit Systems, Iramali 18, P.O. Box 1000, Balzers LI-9496, Liechtenstein; E-Mails: martin.kratzer@oerlikon.com (M.K.); martin-dubs@bluewin.ch (M.D.); heinz.felzer@oerlikon.com (H.F.); robert.mamazza@oerlikon.com (R.M.)

**Keywords:** thin film capacitors, barium titanate, high-k

## Abstract

Simple thin-film capacitor stacks were fabricated from sputter-deposited doped barium titanate dielectric films with sputtered Pt and/or Ni electrodes and characterized electrically. Here, we report small signal, low frequency capacitance and parallel resistance data measured as a function of applied DC bias, polarization *versus* applied electric field strength and DC load/unload experiments. These capacitors exhibited significant leakage (in the range 8–210 μA/cm^2^) and dielectric loss. Measured breakdown strength for the sputtered doped barium titanate films was in the range 200 kV/cm −2 MV/cm. For all devices tested, we observed clear evidence for dielectric saturation at applied electric field strengths above 100 kV/cm: saturated polarization was in the range 8–15 μC/cm^2^. When cycled under DC conditions, the maximum energy density measured for any of the capacitors tested here was ~4.7 × 10^−2^ W-h/liter based on the volume of the dielectric material only. This corresponds to a specific energy of ~8 × 10^−3^ W-h/kg, again calculated on a dielectric-only basis. These results are compared to those reported by other authors and a simple theoretical treatment provided that quantifies the maximum energy that can be stored in these and similar devices as a function of dielectric strength and saturation polarization. Finally, a predictive model is developed to provide guidance on how to tailor the relative permittivities of high-k dielectrics in order to optimize their energy storage capacities.

## 1. Introduction

Barium titanate and its relatives with the perovskite structure have been called the “most significant electroceramic dielectric phase in industry” [1]. These materials are widely used as capacitor dielectrics on account of their high relative permittivity; they are also used as piezoelectrics, pyroelectrics and in electro-optics. In 2000, a United States (US) patent was issued to Peter Hansen that claimed capacitors with dielectrics based on doped barium titanate with reported maximum relative permittivities up to 33,500 [2]. Later, similar materials formed the basis for a series of patents that claimed an ‘Electrical Energy Storage Unit’ (EESU) suitable for electric vehicle applications with an energy density of >300 W-h/kg, significantly higher than the lithium-ion batteries typically used in today’s hybrid-electric and plug-in electric vehicles [3,4,5]. The ability to store such large amounts of energy in what is essentially a very large multi-layer ceramic capacitor (MLCC) depends critically on the ability of the dielectric to maintain a very high relative permittivity at high electric fields. If, as Weir and Nelson claimed in U.S. patent 7,466,536, no evidence for dielectric saturation is in evidence up to electric field strengths of ~3 MV/cm [4], then similar, smaller EESU’s could find application as energy storage capacitors and ‘electronic batteries’ in System-in-Package (SiP) applications.

There is considerable interest in replacing the thick film deposition techniques used for manufacturing today’s MLCCs with thin-film techniques such as sol-gel deposition, chemical vapor deposition (CVD) and physical vapor deposition (PVD); in particular, doped barium titanates deposited by thin-film techniques have been proposed for high frequency capacitors [6]. Other authors have proposed incorporating passive electronic components and rechargeable power storage such as thin-film lithium batteries and thin-film supercapacitors into a multi-function integrated circuit (IC) package in the not-too-distant future [7,8,9]. Such systems could be extremely useful if deployed so as to form a network of autonomous sensors that could monitor the status of numerous parameters around the world, enabling us to detect and combat forest fires, pollution and disease (to name but a few) and even help to predict earthquakes. An energy storage unit with an energy density significantly higher than a thin-film lithium battery, the power density and cycle life of an electrostatic capacitor and that is made from inexpensive, non-hazardous materials such as doped barium titanate and nickel would have a significant impact on the viability of such self-powered sensors that could then operate almost indefinitely using energy harvested from the surrounding ambient by integrated photovoltaic, piezoelectric or thermoelectric devices.

PVD is already well-established in the semiconductor packaging industry. Fabricators that manufacture so-called flip-chip devices typically use a stack of sputtered films as “under bump metal” (UBM) under their solder bumps [10]. Barium titanate is also compatible with semiconductor devices (having been proposed as a capacitor dielectric for memory [11,12]), as is Ni. Consequently, the use of PVD to deposit these materials for subsequent incorporation into a semiconductor package could be conveniently carried out in a packaging foundry or by an integrated device manufacturer (IDM). In contrast, thin-film lithium batteries and/or electrochemical supercapacitors would be better manufactured remotely, perhaps by a third party, since alkali metals can cause serious performance and yield problems should they contaminate complementary metal-oxide-semiconductor (CMOS) or other silicon-based IC production facilities [13].

In an earlier paper, we described an apparatus and method for fabricating simple capacitors with sputtered top and bottom electrodes and sputtered doped barium titanate dielectrics [14]. We also reported relative permittivity and resistivity data derived from small signal capacitance and parallel resistance measurements on these devices at low frequencies. In this work, we report additional electrical characterization data {breakdown voltages (V_bd_), leakage currents, polarization and specific energies} for these thin-film capacitors.

## 2. Experimental Section

The barium calcium zirconate-titanate (BCZTO) dielectric films investigated in this work were sputtered from two different targets, both with an overall composition of Ba_0.96_Ca_0.04_Ti_0.82_Zr_0.18_O_3_, one purchased from SCI Engineered Materials of Columbus, Ohio and the other from Altus Technologies Pte Ltd., Singapore. No significant differences in the resulting film properties due to the change in targets could be discerned. Electrodes were either sputtered Pt or Ni. The procedures for fabricating thin-film capacitors on 4-inch diameter Si substrates using a PVD cluster tool were described in an earlier paper [14]; deposition conditions and film characterization data can be found therein. 3.5 mm diameter capacitor dots were used for breakdown voltage measurements (statistically, they were less likely to incorporate a defect in their structure) while larger 5.5 mm dots were used when attempting to measure the energy density (to provide a larger absolute value). The capacitance and parallel resistance of these thin-film capacitors with ~500 nm thick dielectrics were measured at small signal and low frequency (50 mV@20 Hz) using an Agilent 4284A Precision LCR meter with a test signal frequency range of 20 Hz–1 MHz. From these results, the relative permittivities and resistivities of the modified barium titanate films were calculated: these data were also provided in our earlier paper [14].

Although much attention has been paid to the behavior of barium titanate and related dielectrics at frequencies of 1 kHz and above, if they are to function effectively in electronic batteries, it is also important to characterize their low frequency and DC performance. Here, we report on: (1) additional capacitance and parallel resistance data measured at different applied DC bias fields; (2) polarization *vs*. electric field data acquired using a home-built Sawyer Tower circuit; (3) DC load/unload data determined using a simple electrical circuit; (4) dielectric breakdown measurements; and (5) relative permittivity as a function of applied frequency up to 1 MHz. Capacitance and parallel resistance measurement, polarization and DC load/unload experiments were carried out in the Oerlikon Systems Research and Development Laboratory in Liechtenstein while the dielectric breakdown and relative permittivity *vs*. frequency data were obtained in the U.S. Army Research Laboratories, Adelphi, Maryland.

## 3. Results and Discussion

### 3.1. Capacitance and Parallel Resistance vs. DC Bias

**Figure 1:**
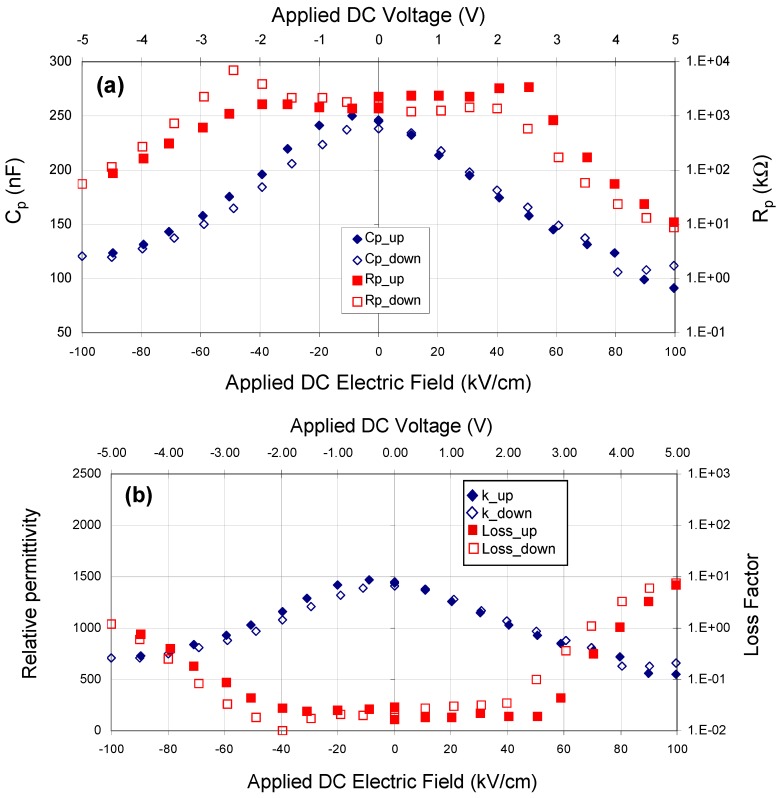
(**a**) Capacitance and resistance and (**b**) relative permittivity and loss factor *vs*. applied DC bias for a test capacitor fabricated with a Pt bottom electrode, a Ni top electrode and a modified barium titanate dielectric deposited at 900 °C, 10 mtorr pressure and a sputter gas mixture of 10% oxygen/ 90% argon. AC probe voltage = 50 mV@20 Hz, dielectric thickness = 500 nm.

Capacitance and parallel resistance data were measured for different applied DC bias fields. For the best capacitors, the capacitance and resistance data were symmetric and reversible: typical behavior is shown in Figure 1a and the relative permittivity and dielectric loss values calculated from these measurements are shown in Figure 1b. The data clearly indicate the onset of dielectric saturation at higher electric fields, with a concomitant increase in loss.

### 3.2. Polarization *vs.* Applied Electric Field

**Figure 2:**
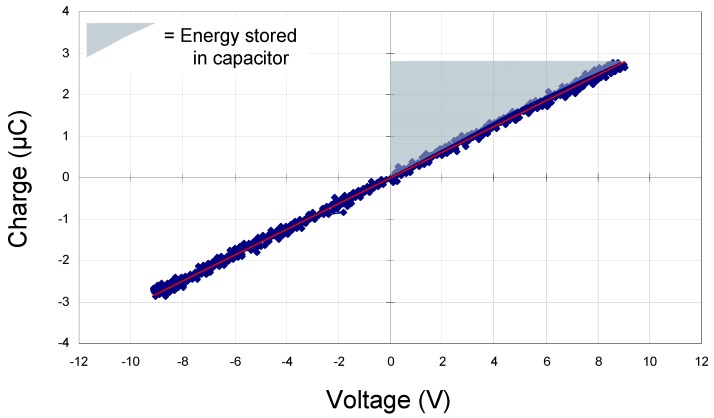
Charge *vs*. voltage for 330 nF standard commercial capacitor. The shaded triangle shows the amount of energy that can be stored in the device (*ʃE·dq*).

**Figure 3:**
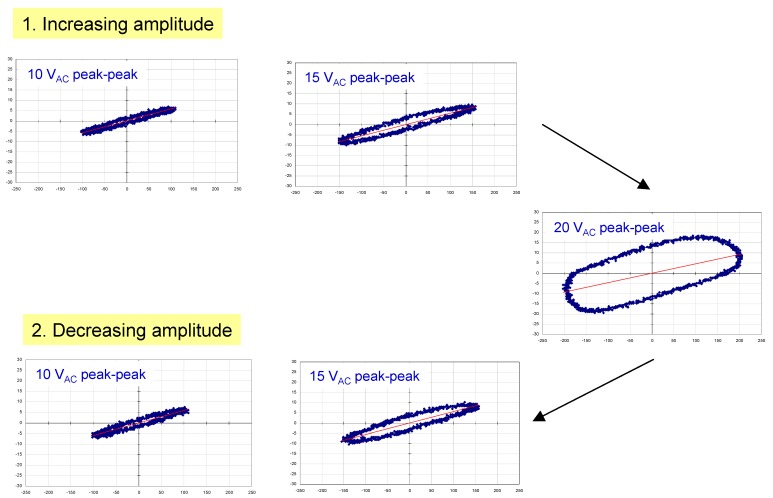
Polarization *vs*. applied voltage hysteresis loops measured at 20 Hz for a capacitor with a Pt bottom electrode, a BCZTO dielectric deposited at 700 °C, 10 mtorr pressure, a sputter gas mixture of 10% oxygen/ 90% argon, and a Ni top electrode.

**Figure 4:**
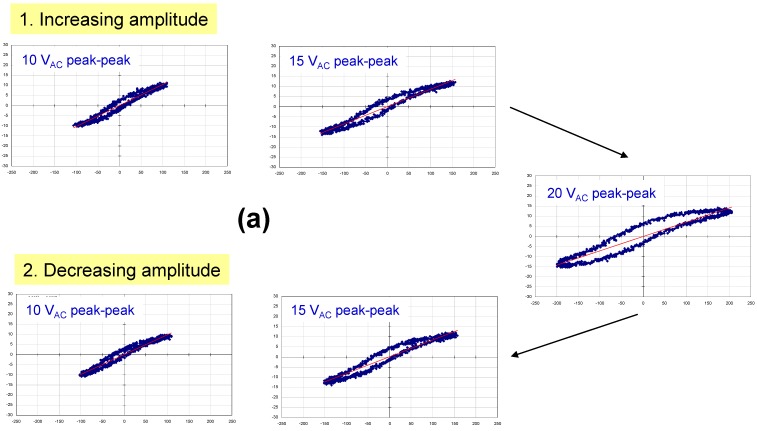

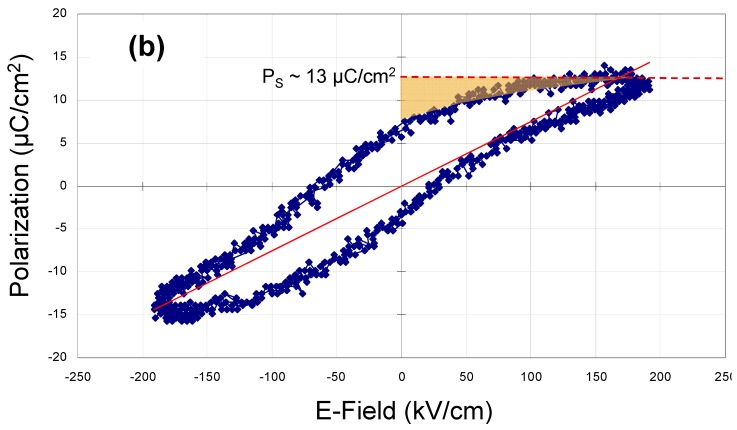
(**a**) Polarization *vs*. applied voltage hysteresis loops measured at 20 Hz for a capacitor with a Pt bottom electrode, a BCZTO dielectric deposited at 900 °C, 10 mtorr pressure, a sputter gas mixture of 10% oxygen/ 90% argon, and a Ni top electrode; (**b**) Blow up of hysteresis loop in (a) at 20 V peak-to-peak.

Polarization *vs*. electric field data were acquired at a frequency of 20 Hz using a home-built Sawyer Tower circuit. Prior to measuring our samples, we tested this using a standard 330 nF commercial capacitor (Figure 2). The area of the shaded triangle shows the amount of energy that can be stored in the device, *ʃE·dq* (since this is a standard commercial capacitor, the value is simply *½QV_app_* or *½CV^2^*). Polarization *vs*. electric field data for two thin-film capacitors with modified barium titanate dielectrics are illustrated in Figure 3 and Figure 4. Figure 3 shows a family of hysteresis loops for a BCZTO sample with a Pt bottom electrode, a dielectric deposited at 700 °C, 10 mtorr pressure, a sputter gas mixture of 10% oxygen/90% argon, and a Ni top electrode. This capacitor exhibited higher dielectric loss than a similar capacitor where the dielectric was sputter deposited at 900 °C (see Figure 1), especially at higher AC amplitudes, although dielectric breakdown was not observed up to 20 V peak-to-peak (for the ~500 nm thick dielectric, this represents a maximum electric field strength of ~200 kV/cm). On reducing the AC amplitude, the residual loss appeared to have increased: we hypothesize that the test current, though not sufficient to cause instant breakdown of the dielectric, did cause internal heating of the device—enough to cause some damage.

Figure 4a shows similar data for a capacitor with lower loss: in this case, the dielectric was deposited at 900 °C—all other parameters were identical to the sample of Figure 3 (where the dielectric was deposited at 700 °C). We hypothesize that the quality of the BCZTO films was improved with higher deposition temperature. Higher oxygen partial pressures in the sputter ambient help to prevent the formation of large numbers of oxygen vacancies in the dielectric that in turn could lead to an increase in conductivity and loss. Figure 4b is a blow up of Figure 4a at maximum applied AC amplitude (20 V peak-to-peak). It illustrates the onset of dielectric saturation at an applied field strength of ~100 kV/cm and also shows how hysteresis loop data can be used to measure the amount of electrical energy that can be stored in these thin-film capacitors (*ʃE·dq* measured as the voltage across the capacitor is decreasing).

The saturated polarization of the sample of Figure 4 (a capacitor with a Pt bottom electrode, a BCZTO dielectric deposited at 900 °C, 10 mtorr pressure, a sputter gas mixture of 10% oxygen/ 90% argon, and a Ni top electrode) was measured as ~13 μC/cm^2^ at a field strength of ~190 kV/cm: this corresponded to a stored energy density of ~0.34 J/cm^3^ or 9.4 × 10^−2^ W-h/liter, calculated based on the volume of the dielectric material only. Using a theoretical bulk density of 6.02 g/cm^3^ for barium titanate from the literature [15], the calculated specific energy was ~1.6 × 10^−2^ W-h/kg, again based on the estimated mass of the dielectric only.

### 3.3. DC Load/Unload Data

**Figure 5:**
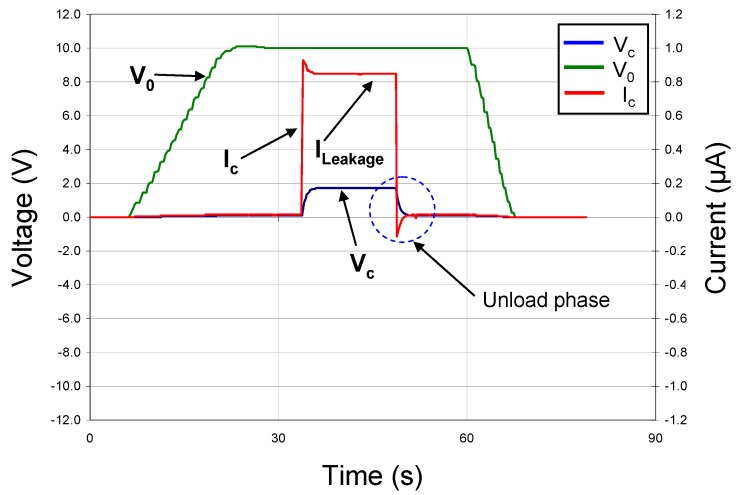
DC charge/discharge test with C = 330 nF (commercial capacitor) & R_Leakage_ = 2 MΩ.

To function as an electronic battery, a capacitor must store and release energy under DC conditions. Consequently, we designed and built a circuit that allowed the device under test to be charged through a standard 10 MΩ resistor to a given voltage with a DC power supply. By monitoring the current in the circuit and the voltage drop across the plates of the capacitor, we were able to measure directly the useful energy stored in our thin-film devices. First, the circuit was tested with a standard 2 MΩ resistor and 330 nF capacitor in parallel to simulate the approximate equivalent circuit of our thin-film capacitors. The resulting DC load/unload data are shown in Figure 5, where the green line represents V_0_, the voltage from the DC power supply, the red line represents I_c_, the current in the circuit and the blue line represents V_c_, the voltage across the capacitor under test. V_0_ was ramped up slowly and a double-throw switch actuated so that the voltage was applied to the capacitor. After an initial spike, I_c_ settled to a steady state value. The switch was then thrown, disconnecting the power supply from the test capacitor and allowing it to discharge through the standard resistor. In Figure 5, this event shows as an instantaneously reversal of I_c_, followed by a slow decay of I_c_ and V_c_ to zero. By integrating the product of V_c_ and I_c_ over time, *i.e*., *ʃV_c_(t)·I_c_(t) dt*, the energy stored in the capacitor was determined.

**Figure 6:**
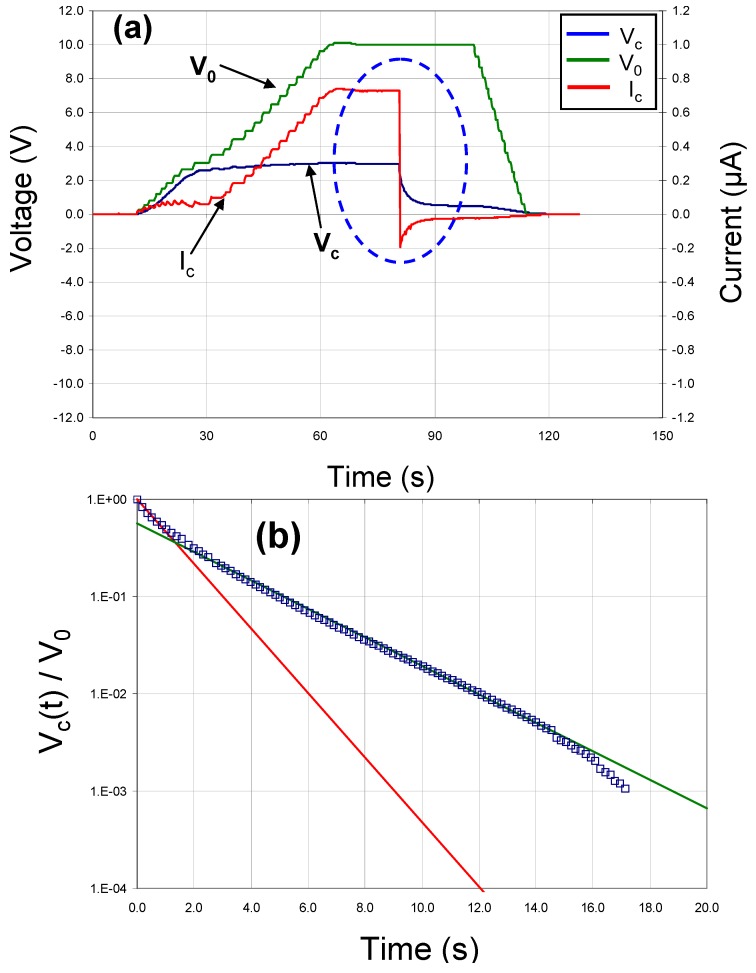
(**a**) DC charge/discharge data and (**b**) voltage decay behavior for capacitor with a Pt bottom electrode, a BCZTO dielectric deposited at 900 °C, 10 mtorr pressure, a sputter gas mixture of 10% oxygen/ 90% argon, and a Ni top electrode.

Figure 6a shows similar data for a test capacitor fabricated with a Pt bottom electrode, a Ni top electrode and a modified barium titanate dielectric deposited at 900 °C, 10 mtorr pressure and a sputter gas mixture of 10% oxygen/ 90% argon. It should be noted that the voltage across this capacitor did not decay exponentially to zero as expected, but rather it appeared that there were two separate discharge mechanisms involved with two different rates (see Figure 6b). This was probably due to domain relaxation.

**Figure 7:**
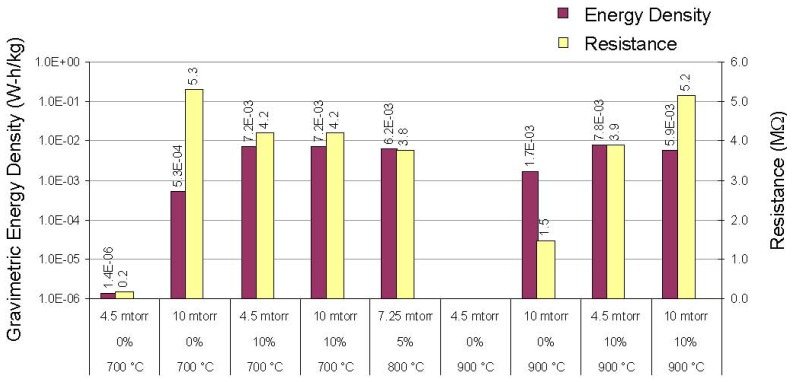
Specific energy and internal resistance of thin-film capacitors *vs*. dielectric deposition conditions.

In all cases, the energy stored in the thin-film capacitors fabricated and tested in this work was very small: the maximum specific energy we observed was ~8 × 10^−3^ W-h/kg (based on the estimated mass of the dielectric only), more than four orders of magnitude less than the values reported by Weir and Nelson. It should also be noted that even the best BCZTO capacitors we tested were too leaky electrically to have any real practical utility as electronic batteries: at an applied voltage of 10 V, leakage currents ranged from ~2–50 μA, corresponding to leakage current densities in the range 8–210 μA/cm^2^, and the maximum internal electrical resistance we observed was ~5 MΩ. Specific energies (based on the estimated mass of the dielectric only) and internal resistances for capacitors with doped barium titanate thin-films deposited at a variety of sputtering conditions are illustrated in Figure 7.

### 3.4. Dielectric Breakdown Measurements

Capacitors were fabricated with Pt top and bottom electrodes as described previously [14]; after patterning the top electrodes, some of these capacitors were subjected to a 1 minute anneal at 350 °C in oxygen. These devices (both annealed and unannealed) were then subjected to increasing AC voltages at 20 Hz until the dielectric failed catastrophically. All of the capacitors tested failed at applied voltages between 25 and >100V, corresponding to electric field strengths in the approximate range 500 kV/cm –2 MV/cm for ~500 nm nominal BCZTO thicknesses. Differences in dielectric breakdown strength could not be correlated to those devices that had been annealed.

### 3.5. Relative Permittivity *vs.* Frequency

Capacitance measurements were performed at frequencies up to 1 MHz on thin-film capacitors with Pt top and bottom electrodes, some of which had been subjected to post-fabrication anneals in oxygen; relative permittivities were then calculated as a function of frequency. ε_r_ remained approximately constant up to 10^5^ Hz before dropping rapidly between 10^5^ and 10^6^ Hz. This drop may be due to the onset of lead resonance: the results might be improved by better shielding. Additional experiments are required to verify this and to optimize the sputtered BCZTO material for high frequency capacitor applications. It is also likely that the processing of the electrodes and the overall structure of the device must be similarly optimized, as this has been found to be very important for other thin-film capacitors that operate at frequencies above 1 MHz [16].

### 3.6. Discussion

Table I tabulates the specific energy calculated from DC load/unload data, the relative permittivity and resistivity of BCZTO from low frequency, small signal capacitance measurements and the dielectric deposition conditions. While higher partial pressures of oxygen in the sputter ambient typically increased the dielectric resistivity, these did not correlate with higher specific energies. Capacitors fabricated from BCZTO films sputtered with 10% oxygen in argon appeared to have the highest specific energies; process temperature in the range 700–900 °C and pressure in the range 4.5–10 mtorr appeared to have minimal effect.

**Table 1:** Specific Energy (from DC load/ unload data), relative permittivity and resistivity (from low frequency, small signal capacitance) *vs*. deposition conditions for thin-film capacitors with sputtered BCZTO dielectric.

P (mtorr)	% O_2_ in Ar	700 °C			800 °C			900 °C		
		Specific Energy (W-h/kg)	ε_r_	R (MΩ)	Specific Energy (W-h/kg)	ε_r_	R (MΩ)	Specific Energy (W-h/kg)	ε_r_	R (MΩ)
4.5	0%	1.4 E-06	2,037	0.2						
	10%	7.2 E-03	962	4.2				1.7 E-03	1,660	1.5
7.25	5%				6.2 E-03	1,278	3.8			
10	0%	5.3 E-04	1,018	5.3				7.8 E-03	1,472	3.9
	10%	7.2 E-03	926	4.2				5.9 E-03	1,489	5.2

To be effective as practical electronic batteries, electrical leakage is a key parameter: even the least leaky of the capacitors tested here discharged much too rapidly for use as a battery. However, it is conceivable that this could be addressed by one or more of several techniques. One possibility would be the addition of a very high resistivity material to the dielectric as advocated by Weir and Nelson. While this would drastically reduce the overall leakage, the disadvantage is that it would have the effect of adding a second capacitor in series, resulting in a drop in total capacitance and electrical energy stored. MLCCs are typically fired in oxygen-containing ambients to anneal out oxygen vacancies that act as electron donors and increase the conductivity of the dielectric films. Controlled addition of donor and/or acceptor atoms in the perovskite lattice can also serve to increase the resistivity. However, such methods typically do not permit MLCCs to retain a high state of charge for hours or days as would be required for them to function effectively in electronic battery applications.

The energy densities and specific energies measured for the thin-film capacitors fabricated in this work were small. Partly, this can be attributed to the fact that only relatively low electric fields were applied; though typically, breakdown strengths were above 200 kV/cm, films stressed at these fields appeared to degrade with repeated cycling. However, even at these relatively low field strengths, there was clear evidence for dielectric saturation in the doped barium titanate films that would serve to limit the maximum energies that could be stored in these devices. It could be argued that this behavior was an artifact peculiar to these sputtered thin-films. Subsequent work conducted at the University of Halle-Wittenberg on capacitors with dielectric composites similar to those reported by Weir and Nelson showed qualitatively similar saturation to the results reported here [17]: a simple theoretical analysis can shed light on this phenomenon. 

For a planar capacitor where the dielectric remains linear up its breakdown strength, the stored energy per unit volume of dielectric (expressed in Joules per cubic meter) is given by:

(1) $$ED=\frac{1}{2}\varepsilon_{0}\cdot \varepsilon_{r}\cdot V^{2}$$

where ED is the volumetric energy density, ε_0_ is the permittivity of free space (=8.854 × 10^−12^ F/m), ε_r_ is the relative permittivity and V is the potential difference in volts across the capacitor plates. If ε_r_ remains linear up to the breakdown voltage of the dielectric, the maximum energy density (*i.e*., energy per unit volume) that can be stored in such a capacitor is limited by the dielectric strength and is given by:

(2) $$ED_{\max}=\frac{1}{2}\varepsilon_{0}\cdot \varepsilon_{r}\cdot {\left(V_{bd}\right)}^{2}$$

here, ED_max_ is the maximum volumetric energy density and V_bd_ is the breakdown voltage of the (linear) dielectric. For a given V_bd_, the energy density of such a capacitor (with a linear dielectric) is directly proportional to ε_r_. Most insulating materials have relative permittivities between 1 and 100. For thin-film capacitors with SiO_2_ or Al_2_O_3_ dielectrics that are reported to have very high breakdown strengths (>5 MV/cm) this sets a practical upper limit on the energy density of 3.1 and 6.7 J/cm^3^ for relative permittivities of 3.9 and 8.4, respectively, at an applied electric field strength of 3 MV/cm. Ta_2_O_5_, with a relative permittivity of 26, would have an energy density of 21 J/cm^3^ if it could withstand a similar electric field. These energy densities correspond to 0.86, 1.86 and 5.83 W-h/liter, respectively. For a hypothetical dielectric with a relative permittivity of 100 that is linear up to an electric field strength of 3 MV/cm, the corresponding energy density would be 22.14 W-h/liter, substantially below the values claimed by Weir and Nelson [3] for capacitors using doped barium titanate dielectrics with relative permittivities up to 33,500 and also significantly below the energy densities of rechargeable lithium-ion and thin-film lithium batteries [18]. It is therefore instructive to consider how additional energy can be stored in polarizable materials such as barium titanate and its relatives with the perovskite structure.

In the cubic perovskite structure of barium titanate, the Ti^4+^ ion is situated on an octahedral site surrounded by six O^2−^ ions. This site is slightly too large for the Ti^4+^ ion and consequently, it can move within this site. In contrast, Ba^2+^ and O^2−^ ions remain essentially fixed in the crystal structure. Because the Ti ion carries a (positive) charge, it will move in response to an imposed external electric field; when the imposed electric field is removed, it will return to its equilibrium position. Below the Curie point where the Ti^4+^ ions are able to occupy more than one non-equivalent site in the oxygen octahedra, this phenomenon gives rise to the observed ferroelectric properties of barium titanate. Above its Curie point, barium titanate has a cubic perovskite structure and behaves as a dielectric with a very large relative permittivity. The cubic perovskite structure of barium titanate allows every Ti^4+^ ion to move a maximum of ~0.25 Å from its equilibrium position.

Using the well-known expression for the work (W) performed by a charged particle moving in an electric field (E):

*W* = *q* × *E* × *δ* (3)

where δ is the distance moved, it can be calculated that, for a field strength of 3 MV/cm given by Weir and Nelson [3,4], the maximum work associated with polarizing cubic barium titanate is ~2.9 kJ/mol. Using a theoretical density of 6.02 g/cm^3^ for barium titanate from the literature [15] and a relative molar mass of 233, we calculate a value of ~3.45 W-h/kg for the specific energy and a value ~21 W-h/liter for the energy density, based on the mass and volume, respectively, of the dielectric material only. If the polarization/depolarization process is 100% efficient and reversible, this figure represents the maximum amount of energy that can be stored in the dielectric due to polarization, absent any quantum mechanical effects. Note that this calculation does not consider the weight and volume of the electrodes, nor does it take into account any packaging that would be required to manufacture a practical device. Also note that this energy density is quantitatively similar to the amount of energy that would be stored in a capacitor with a linear dielectric of relative permittivity 100 at an electric field strength of 3 MV/cm. It cannot account for the large difference between the energy density calculated for a planar capacitor with a hypothetical dielectric that is linear up to 3 MV/cm and the values claimed by Weir and Nelson for their EESU [3,4,5].

In order to increase the maximum amount of energy that can be stored by polarization of Ti^4+^ in cubic barium titanate, it is necessary to increase the breakdown strength. While in the literature, there have been reports of dielectric breakdown strengths of 10 MV/cm and slightly above for some materials [19], in general, applying an electric field that is close to its breakdown strength causes damage to a capacitor dielectric. Consequently, we hypothesize that it is not possible to achieve anywhere close to the specific energies of >300 W-h/kg at 3 MV/cm as claimed by Weir and Nelson for capacitors based on modified barium titanate. Indeed, it would appear that fundamental limitations (typically thought of as dielectric saturation) limit specific energies to approximately one order of magnitude less than these values.

**Figure 8:**
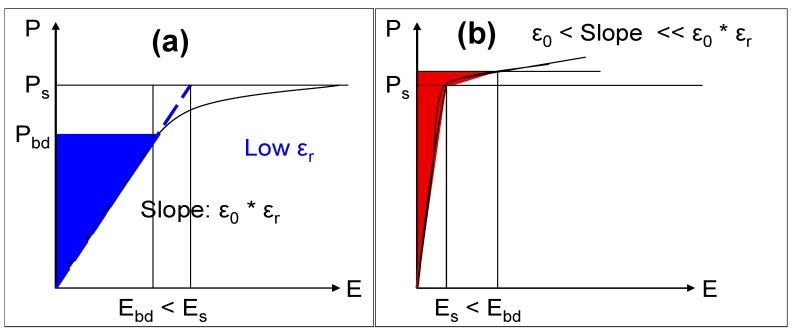
(**a**) Schematic of polarization *vs*. electric field strength for case where stored energy (blue shaded area) limited by dielectric breakdown (E_bd_ < E_s_); (**b**) As for (**a**) but here stored energy (red shaded area) is limited by saturation polarization (E_s_ < E_bd_).

As a general rule, capacitors fabricated with dielectrics that have the highest relative permittivities will not have the highest energy densities: this can only be achieved by matching ε_r_ and V_bd_ appropriately. Consider a planar capacitor with a hypothetical dielectric that is linear up to its breakdown field: a graphical representation of the polarization *vs*. the electric field strength for such a device is shown schematically in Figure 8a. Here, the maximum energy density of the capacitor can be calculated according to Equation (2). The case for a hypothetical dielectric where the relative permittivity is large and varies approximately linearly with applied electric field strength up to its saturation polarization and thereafter drops substantially until V_bd_ is reached, is shown schematically in Figure 8b. In this case, the maximum energy density of the capacitor will be primarily constrained by the saturation polarization of the dielectric and thus, it is convenient to express the energy density of such a capacitor in terms of the polarization:

(4) $$ED_{\max}≅\frac{1}{2}P_{s}^{2}\cdot {\left(\varepsilon_{0}\cdot \varepsilon_{r}\right)}^{-1}$$

where P_s_ is the saturation polarization. Examining Equations (2) and (4), where the maximum energy density is limited by the breakdown voltage of the dielectric (Equation (2)), it is proportional to ε_r_, the relative permittivity of the dielectric. However, where the maximum energy density is limited by the saturation polarization of the dielectric (Equation (4)), it is inversely proportional to ε_r_. In this case, increasing the relative permittivity of the dielectric will result in a lower maximum energy density.

**Figure 9:**
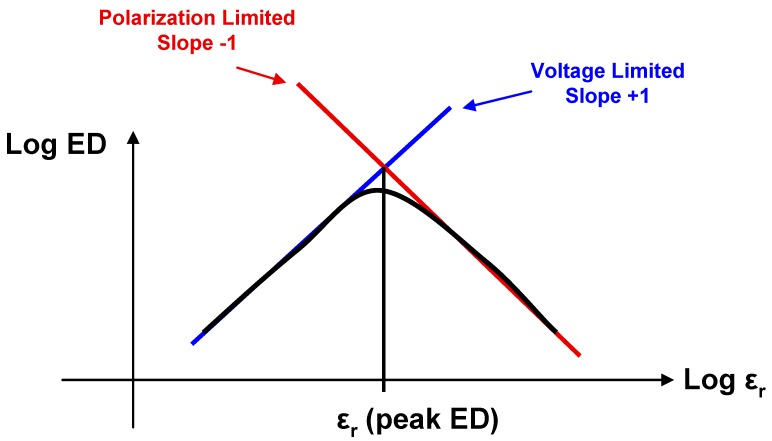
Plot of log energy density *vs*. log relative permittivity for a family of materials with a set polarization and breakdown voltage.

Usually, the higher the relative permittivity of a dielectric material, the more likely it is to saturate below its breakdown strength. Therefore Equation (2) will hold for relatively low ε_r_ materials while Equation (4) will apply to materials with large ε_r_. As a result of this analysis, we can therefore conclude that for a family of linear dielectric materials with a given breakdown voltage and polarization, the maximum energy density for a capacitor using these materials will occur where dielectric breakdown and saturation polarization coincide. This is shown schematically in Figure 9, where the logarithm of the energy density of such a capacitor (*log ED*) is plotted *vs*. log ε_r_. At low ε_r_, the slope for *log ED* *vs*. log ε_r_ is 1, while at high ε_r_, the slope for *log ED* *vs*. log ε_r_ is −1. At the point of intersection (at an intermediate value of ε_r_) *log ED* is a maximum.

Typical high-k materials are not linear and therefore this principle can only serve as an approximate guide when attempting to tailor ε_r_ to maximize the energy storage of a capacitor. However, it does highlight the fact that attempts to boost the maximum energy density of electrostatic capacitors by designing relaxor ferroelectric materials with extremely high relative permittivities are futile. Such materials would saturate at relatively low electric fields, well below the breakdown voltage of the dielectric. Once dielectric saturation has occurred, the rate at which stored energy increases with additional applied electric field strength across the capacitor is small.

**Figure 10:**
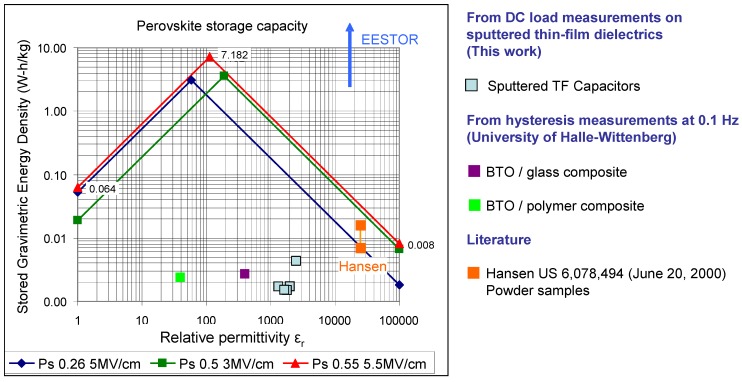
Plot of log energy density *vs*. log relative permittivity for modified barium titanates with different assumed polarization (P_s_) and breakdown voltages. Also shown are thin-film (TF) capacitor data measured in this work, barium titanate (BTO) composite data from the University of Halle-Wittenberg and data from the literature (Hansen [2]).

Figure 10 shows several plots for different assumed polarizations and breakdown strengths typical of barium titanate-based relaxor ferroelectrics. Also included in Figure 10 are data measured in this work, by the University of Halle-Wittenberg [17] and those of Hansen taken from the literature [2]. As can be seen, the data claimed by Weir and Nelson [3,4,5] are off the chart by over an order of magnitude. Referring to Figure 10, in order to achieve the maximum specific energy in an electrostatic capacitor based on modified barium titanate dielectrics, the optimum relative permittivity is predicted to be in the range from ~60 to ~200. However, certain practical considerations dictate that the optimum ε_r_ should be higher for most applications. This is because when stressed at electrical field strengths close to breakdown, most dielectrics experience some slight damage; repeated cycling eventually causes breakdown at field strengths well below their rated dielectric strength. Typically, ceramic capacitors should be designed with a built-in safety factor and operated significantly below the maximum breakdown field of the dielectric [20]. Consequently, the optimum energy storage characteristics of electrostatic capacitors based on modified barium titanate dielectrics will be at slightly higher relative permittivities than those predicted by Figure 10. However, owing to the need to limit the electric field strength, the maximum practical specific energy and energy density that can be achieved in this family of capacitors will be lower (by between 10-100x) than the ~3.45 W-h/kg and ~21 W-h/liter quoted earlier for materials with breakdown strengths in the range 3–5 MV/cm; similar materials with breakdown strengths as high as 10 MV/cm have been reported in the literature [19] but even these materials will likely have maximum practical and theoretical specific energies significantly below today’s lithium-ion batteries. It appears that, in the absence of some yet-to-be-discovered quantum effect, electrostatic capacitors based on relaxor ferroelectrics are not suitable replacements for electrochemical batteries in applications that primarily require high energy density.

## 4. Conclusions

In an earlier paper, we described the Oerlikon Multisource Quattro (MSQ) compact co-sputtering source and very hot rotating chuck combination and showed its capacity to deposit high-k dielectrics based on barium titanate in their desired perovskite structure above 600 °C. Simple single layer capacitors were fabricated by sequentially depositing bottom electrode, dielectric and top electrode in a cluster tool without breaking vacuum. X-ray diffraction data and small signal, low frequency measurements of the relative permittivity and resistivity were presented [14].

In this paper, we extended the small signal, low frequency measurements to show how capacitance and loss were influenced by large applied DC fields. We also reported polarization and DC load/unload data for the thin-film capacitors, from which energy densities and specific energies were derived. Strong evidence for dielectric saturation was observed well below the typical breakdown strength of >200 kV/cm.

We described a simple theoretical approach to quantify the maximum energy that can be stored in electrostatic capacitors that use polarizable high-k dielectrics based on barium titanate and similar perovskite phases as a function of dielectric strength and saturation polarization. We also developed a predictive model to provide guidance on how to tailor the relative permittivities of these high-k dielectrics in order to optimize their energy storage capacities.

## References

[1] Bhalla A.S., Guo R., Roy R. (2000). The perovskite structure—A review of its role in ceramic science and technology. Mat. Res. Innovat..

[2] Hansen P. (2000). Multilayer capacitor comprising barium-titanate doped with silver and rare earth metal. U.S. Patent.

[3] Weir R.D., Nelson C.W. (2006). Electrical-energy-storage unit (EESU) utilizing ceramic and integrated-circuit technologies for replacement of electrochemical batteries. U.S. Patent.

[4] Weir R.D., Nelson C.W. (2008). Utilization of poly(ethylene terephthalate) plastic and composition-modified barium titanate powders in a matrix that allows polarization and the use of integrated-circuit technologies for the production of lightweight ultrahigh electrical energy storage units (EESU). U.S. Patent.

[5] Weir R.D., Nelson C.W. (2009). Electrical-energy-storage unit (EESU) utilizing ceramic and integrated-circuit technologies for replacement of electrochemical batteries. U.S. Patent.

[6] Randall M., Skamser D., Kinard T., Qazi J., Tajuddin A., Trolier-McKinstry S., Randall C., Ko S.W., Dechakupt T. (2007). Thin film MLCC. CARTS USA 2007 Symposium Proceedings.

[7] Torres E.O., Rincón-Mora G.A. (2005). Long-lasting, self-sustaining and energy-harvesting system-in-a-package (SiP) wireless microsensor solution. Presented at International Conference on Energy, Environment, and Disasters (INCEED).

[8] Torres E.O., Rincón-Mora G.A. (2008). Energy-harvesting system-in-package (SiP) microsystem. ASCE J. Energy Eng..

[9] Tummala R.R. (2010). Packaging of 3D ICs to 3D systems. Presented at Workshop on 3D Wafer Level Packaging, ESTC 2010.

[10] Keigler A., Golovato S., O’Donnell K., Chiu J., Hollman R. (2007). PVD processing for flip chip: Considering adhesion layer properties. Advanced Packaging.

[11] Yeh M.H., Liu Y.C., Liu K.S., Lin I.N., Lee J.Y.M., Cheng H.F. (1993). Electrical characteristics of barium titanate films prepared by laser ablation. J. Appl. Phys..

[12] Lee M.-K., Liao H.-C., Tung K.-W., Shih C.-M., Shih T.-H. (2002). Liquid-phase-deposited barium titanate thin films on silicon. J. Phys. D Appl. Phys..

[13] Gorodokin V., Zemlyanov D., Sergienko A. (2004). Metallic contamination in silicon processing. Proceedings of the 2004 23rd IEEE Convention of Electrical and Electronics Engineers in Israel.

[14] Reynolds G.J., Kratzer M., Dubs M., Felzer H., Mamazza R. (2012). Sputtered modified barium titanate for thin-film capacitor applications. Materials.

[15] Chen C-Y, Tuan W-H. (2000). Effect of silver on the sintering and grain-growth behavior of barium titanate. J. Am. Ceram. Soc..

[16] Xu H, Pervez N.K., Park J.H., Sanabria C., York R.A. (2005). Tunable microwave integrated circuits using BST thin film capacitors with device structure optimization. Presented at The 17th International Symposium on Integrated Ferroelectrics.

[17] Beige H., Ebbinghaus S., Grossman T., Ehrhardt C., Leipner H.S., Fuhrmann B., Diestelhorst M., Pientschke C. (2010). Feasibility Study for Energy Storage in Nanopowders.

[18] Broussely M., Nazri G.-A., Pistoia G. (2003). Li-Ion batteries for EV, HEV and other industrial applications. Lithium Batteries: Science and Technology.

[19] Naidu M.S., Kamaraju V. (2008). Breakdown in solid dielectrics. High Voltage Engineering.

[20] Herbert J.M. (1985). Ceramic Dielectrics and Capacitors.

